# Understanding how communities respond to COVID-19: experiences from the Orthodox Jewish communities of Antwerp city

**DOI:** 10.1186/s12939-021-01417-2

**Published:** 2021-03-15

**Authors:** Jef Vanhamel, Marie Meudec, Ella Van Landeghem, Maya Ronse, Charlotte Gryseels, Thijs Reyniers, Anke Rotsaert, Charles Ddungu, Lazare Manirankunda, Deogratias Katsuva, Koen Peeters Grietens, Christiana Nöstlinger

**Affiliations:** 1 Department of Public Health, Institute of Tropical Medicine, Nationalestraat 155, B-2000 Antwerp, Belgium; 2 Outbreak Research Team, Institute of Tropical Medicine, Nationalestraat 155, B-2000 Antwerp, Belgium; 3 School of Tropical Medicine and Global Health, Nagasaki University, Nagasaki, Japan; 4 Amsterdam Institute for Social Science Research, Amsterdam, The Netherlands

**Keywords:** COVID-19, Community engagement, Orthodox Judaism, Participatory approach

## Abstract

**Background:**

The importance of community involvement in the response against disease outbreaks has been well established. However, we lack insights into local communities’ experiences in coping with the current COVID-19 pandemic. This study explored both the impact of, and response to, COVID-19 within the Orthodox Jewish communities of Antwerp (Belgium) during the first lockdown period (March 2020 – May 2020).

**Methods:**

We conducted an explorative qualitative study using a participatory approach. First, we performed a community mapping to identify relevant stakeholders. Through the active involvement of a community advisory board and based on qualitative interviews with key-informants and community members, we elicited lived experiences, attitudes, and perceptions towards COVID-19. Interviews were conducted both face-to-face and using online web conferencing technology. Data were analyzed inductively according to the principles of thematic analysis.

**Results:**

Government-issued outbreak control measures presented context-specific challenges to the Orthodox Jewish communities in Antwerp. They related mainly to the remote organization of religious life, and practicing physical distancing in socially and culturally strongly connected communities. Existing community resources were rapidly mobilized to adapt to the outbreak and to self-organize response initiatives within communities. The active involvement of community and religious leaders in risk communication proved to be of great importance to facilitate the coverage and uptake of pandemic control measures while protecting essential community values and traditions. Creating bottom-up and community-adapted communication strategies, including addressing language barriers and involving Rabbis in the dissemination of prevention messages, fostered a feeling of trust in government’s response measures. However, unmet information and prevention needs were also identified, such as the need for inclusive communication by public authorities and the need to mitigate the negative effects of stigmatization.

**Conclusion:**

The experiences of Orthodox Jewish communities in Antwerp demonstrate a valuable example of a feasible community-centered approach to health emergencies. Increasing the engagement of communities in local decision-making and governance structures remains a key strategy to respond to unmet information and prevention needs.

## Background

Engaging communities in health interventions is deemed crucial to improve population health and well-being, also in emergency contexts [1–4]. The World Health Organization (WHO) therefore actively recommends community participation in disease control strategies [5]. The expertise of community members is rooted in local realities and in their historical context. Their insights might therefore contribute to a better understanding of certain social structures, community norms, and belief systems that might otherwise remain inaccessible or unknown to ‘outsiders’, unless explicitly researched [6]. Understanding how these local realities interact with meso- and macro-societal structures is paramount to developing context-specific and tailored solutions that will facilitate the implementation and adoption of health interventions [7].

In early 2020, the rapid spread of a newly identified coronavirus, SARS-CoV-2, led many countries to take unprecedented outbreak control measures to mitigate the otherwise devastating effects of this pandemic on individual and public health outcomes. In the absence of effective pharmaceutical interventions, these measures relied heavily on reducing close contact between people (e.g. physical distancing, stay-at-home recommendations, closure of public spaces etc.). The impact of this epidemic on social life in communities therefore cannot be underestimated. Despite the documented benefits of a bottom-up and community-based approach in disease outbreaks, knowledge of the engagement of local communities in either the design, planning, or delivery of preventive measures in the current COVID-19 pandemic remains limited [8–11]. Yet, communities consist of individuals with agency, able to respond and adapt to crisis situations by building on existing strengths and resources that are readily available to them. We currently lack insight into local communities’ experiences in dealing with the COVID-19 pandemic and coping with COVID-19 control measures. Such evidence might comprise useful examples and lessons that can inspire future initiatives and creative solutions for outbreak prevention and preparedness in different contexts.

In Belgium, Antwerp city is home to a very diverse population of heterogeneous social, cultural, ethnic and religious communities. This includes one of Europe’s largest communities of Orthodox Jews. The Jewish communities in Antwerp are currently estimated at about 25,000 members. The socio-demographic profile of current Antwerp Jewish inhabitants is mainly influenced by the ethnic background of returning survivors following the Holocaust in Europe during the Second World War [12]. A large proportion consisted of Eastern European Jews from different Orthodox and Haredi traditions, fleeing anti-Semitism in their countries of origin (see Table 1 for an overview of the different Jewish communities and identities in Antwerp) [13]. Over time, organized Jewish life in Antwerp became increasingly religious-orthodox, with a heterogeneous landscape of different Jewish religious communities maintaining a variety of cultural identities and traditional ways of life, while at the same time living integrated within the majority society in the Belgian context [12].

**Table 1:** Overview of the Jewish communities of Antwerp

**Most common Jewish identities among members of Antwerp Jewish communities**	
Haredi	While Brussels hosts the largest Jewish community in Belgium, Antwerp is known to be home to one of Europe’s largest communities of Haredi Jews (sometimes also referred to as ‘ultra-Orthodox’ Jews). About one third of all Jewish people living in Antwerp are estimated to belong to this group [13].
	One important characteristic of the Haredim is a strict adherence to a specific body of Jewish religious laws called the *Halacha* [14]. These rules strongly organize personal, social and spiritual life in Haredi communities, with reliance on religious leaders (or ‘teachers’) to provide spiritual guidance. These leaders also are well-positioned to advise whether certain practices, under certain circumstances, can be considered *kosher* (i.e. in line with the *Halacha*) or not.
	The Haredim symbolically manifest the cultural boundaries of their group by a very distinctive dress code, making them a more segregated and visually identifiable group. Men are often bearded, wear a *kipah* (with or without *shtreimel*), and cover themselves with long black coats. Haredi women are equally expected to dress modestly, wear skirts, and cover their heads (e.g. with a *sheitel*) [13].
	The community of Haredi Jews consists of many different sub-groups. One of the largest movements is the Hassidic one, comprising different communities (or ‘courts’) based on their geographical origin (e.g. the Hassidic community of *Belz, Satmer, Vishnitz* etc.) [15]. The community of *Belz* is numerically the largest Hassidic community in Antwerp.
Orthodox	The term ‘Orthodox’ is used to refer to those who identify themselves as Jewish in a rather conservative way when it comes to the observance of Jewish religious rules. Yet in general, the Orthodox community adopts a more pragmatic and liberal attitude compared to the Haredi group, leaving more space for flexibility and compromise in the application of religious rules in daily life [16].
	The limits and boundaries of what constitutes Orthodox Judaism is a matter of debate, as there is an inherent plurality of different Orthodox sub-groups and communities, each having their own unique cultural and religious heritage [17]. Often the term ‘Orthodox’ is used as a popular term encompassing both Haredi and (other) Orthodox Jewish communities.
Secular/traditional	Some community members feel their Jewish identity is not linked to religious faith per se. In the first place, they feel connected through the various common cultural and family traditions that, to them, comprise the feeling of ‘being Jewish’. Rather than experiencing religious obligations, they may participate in religious activities as part of celebrating this tradition. However, others might feel more secular, or even anti-religious [18].
**Organization of the Antwerp Jewish communities**	
Shomre Hadas	The largest formal Jewish organization in Antwerp is called Shomre Hadas [13]. Most of its members belong to the above described groups of Orthodox and traditional Jews. Each community organizes religious services, maintains their synagogues, provides appropriate burial services, and observes the rules of *kosher* food preparation.
Machsike Hadas	The Machsike Hadas is a somewhat smaller and in general more conservative organization compared to the Shomre Hadas. It mainly consists of strictly Orthodox and Haredi communities. Also most of the Hassidic groups fall under this larger administrative community.
Sephardic community	This is the smallest Jewish community in Antwerp, which historically has its origins in Portugal.

Since the beginning of the pandemic, popular media worldwide have reported on Orthodox Jewish communities, often depicting them as particularly vulnerable to, or even responsible for, widespread COVID-19 transmission because of socio-cultural dynamics and a specific Orthodox Jewish lifestyle [19–21]. However, in Belgium, stratified epidemiological data on the COVID-19 burden among cultural, ethnic, or religious minority populations are not available. Therefore, information on the burden and spread of COVID-19 in the Orthodox Jewish communities of Antwerp city consists mainly of anecdotal evidence. There is a growing body of evidence showing that ethnic, racial and cultural minority populations have been disproportionally affected by the current COVID-19 pandemic and its direct or indirect consequences. The reasons are complex, multi-dimensional, and frequently intertwined with socio-economic vulnerabilities often more prevalent among such groups [22–27]. Framing specific minority communities as being particularly implicated in disease outbreaks might be stigmatizing, be perceived by community members as stigmatizing, and contribute to discrimination and fear [28, 29]. Given that Antwerp hosts a great diversity of minority communities, understanding and identifying their essential information and prevention needs will be of great importance to develop community-centered, culturally-sensitive health interventions to respond to those needs, as well as to prevent and mitigate the effects of stigmatization.

In this study, we aimed to understand the impact of COVID-19 on the Orthodox Jewish communities of Antwerp city during the first ‘lockdown’ period between March and May 2020. We explored how local response mechanisms and coping strategies were shaped and developed within their local context, and how these relate broader cultural, religious, and socio-economic factors. This allowed us to identify unmet information and prevention needs in such minority communities and how they could be addressed by future interventions.

## Methods

### Study background and design

The qualitative explorative study on which we report here is part of a larger and ongoing assessment of the impact of and response to COVID-19 during the first lockdown period on different migrant, ethnic, and religious minority communities living in Antwerp, Belgium. The main objective of the overall research study was to rapidly assess the information and prevention needs among such communities (phase 1) in order to co-create and implement tailored prevention and communication measures where needed and desired (phase 2). We therefore adopted an iterative and inductive study approach, emphasizing the community context as the research setting. In doing so, we sought to stimulate a horizontal dialogue with communities, facilitating stakeholder collaborations, and building trust [30]. In this paper, we exclusively report on our findings of phase 1 of the research within the Orthodox Jewish communities.

Three local community networks participated in the overall research: members of a network of sub-Saharan African migrant communities, members of communities with various migration backgrounds (e.g. Moroccan, Turkish and Syrian), and the Orthodox Jewish communities. The choice to engage with these particular communities was both purposive and pragmatic, based on emergent routinely available ecological data showing that some geographical districts home to superdiverse populations were disproportionally affected by COVID-19 [31]. As there are no Belgian epidemiological data stratified by religion or cultural-ethnic indicators available, geographical precedence of cases was the main source of information. Moreover, previous research has established the increased vulnerability of minority communities to unfavorable health outcomes in the context of infectious disease outbreaks [32, 33].

To ensure collaboration with all relevant stakeholders, and to obtain constant input and feedback at each step of the research, a community advisory board (CAB) was set up. This platform included representatives of relevant community- and faith-based organizations of the involved communities, as well as delegates from the local authorities at a city-level. This allowed for a short line of communication between the on-ground community experiences and a higher policy level where decisions were made.

In a first preparatory stage of this study within the network of Orthodox Jewish communities, we used a community mapping, based on existing information networks to identify key informants. In a second stage, we interviewed these key informants to (1) better understand community structures and dynamics, (2) to have an initial assessment of the communities’ responses towards the outbreak, and (3) to build trust and rapport. In a third stage, we interviewed community members in general to elicit lived experiences, perceptions, attitudes, and remaining information and prevention needs related to COVID-19.

### Data collection

*Community mapping:* we reviewed academic and grey literature on the Antwerp Jewish community, and we scanned local community websites and social media platforms to identify relevant community leaders and organizations. In addition, we held informal conversations by e-mail and telephone with purposively selected informants within our existing information networks, based on their background and relevant expertise with regard to the Orthodox Jewish communities of Antwerp. This mapping exercise led to the identification of relevant stakeholders to be included for subsequent key informant interviews. Members of the CAB were crucial in identifying additional key informants and helped to establish initial contact between the researcher performing the data collection (JV) and informants from within their respective communities.

*Key informant interviews* were guided by a topic guide, covering the following main themes: community structure; impact of COVID-19 on community organization; access to information (in general, and on COVID-19); community responses towards COVID-19, as well as remaining information and prevention needs. We asked interviewees if they could provide us with contacts of other community members for subsequent in-depth interviews. This ‘snowballing’ method, whereby previous participants could serve as a point of reference for subsequent interviewees, has shown to aid the development of a form of trust and rapport when researchers are perceived as ‘outsiders’ to the group they are trying to reach [34].

*In-depth interviews* were equally guided by a topic guide, focusing on subjects related to community members’ personal experiences of COVID-19 and its impact on their lives and their communities; on risk perception towards COVID-19;on accessing information related to COVID-19; and on perception towards prevention and control measures.

In order to respect COVID-19 prevention measures related to physical distancing, web conferencing technology was used during the strict lockdown period, followed by face-to-face interviews with physical distancing as soon as measures were loosened. ‘Zoom’ was used as a videoconferencing platform, with an institutional license and in compliance with General Data Protection and Regulation (GDPR).

### Data analysis

All interviews were audio-recorded, with verbal consent of the participants, and subsequently combined with the researcher’s notes into extensive summaries. Data were analysed inductively using QSR-Nvivo (release 1.3, March 2020) according to the principles of thematic analysis [35]. In a first phase, overarching themes related to topic guide questions were outlined. A data-driven codebook was then established based on an analysis of the data corresponding with the overarching themes. Sub-themes and additionally emerging descriptive codes were assigned upon a re-analysis of the data by the first author. Inconsistencies or unclarities in the interpretation of the data were addressed through discussions with members of the research team and the CAB.

## Results

We conducted 15 interviews between April 24, 2020 and June 25, 2020. All participants but one were male and aged between 30 and 75 years. Six interviews were conducted with a total of seven key informants (one qualified as a ‘duo interview’). These key informants were mainly members of the Antwerp Jewish communities with a leadership or coordinating function within the community (see Table 2). In addition to the key informant interviews, we also conducted nine in-depth interviews with members of the Antwerp Jewish communities that had no particular leading role in the community, and were more involved in receiving rather than coordinating information flows related to COVID-19. Even though community members participated who both did and did not self-identify as Orthodox and Haredi Jewish, all interviews focused on participants’ experiences with, and perceptions of, Antwerp Orthodox Jewish communities, hereby including Haredi groups.

**Table 2:** Characteristics of study participants

Profile of participants	Number included
Key informant interviews	
Leaders of community- and faith-based organizations	4
Health professional working in a practice serving Antwerp Orthodox Jewish communities	1
Academic lecturer with a professional background in Jewish studies and philosophy	1
Civil servant at the local authorities (city-level)	1
In-depth interviews	
Community members who identified as Orthodox	2
Community members who identified as Haredi	3
Community members who identified as traditionally Jewish	4

Three main overarching themes emerged from the data; (1) the impact of COVID-19 and related control measures on the Antwerp Orthodox Jewish communities, (2) the communities’ responses to COVID-19, and (3) unmet information and prevention needs.

### Impact of COVID-19 and related control measures on Antwerp orthodox Jewish communities

#### Religious life and the orthodox Jewish identity

All participants acknowledged that the current coronavirus pandemic posed a unique challenge to the practice of religion for Orthodox and Haredi Jews. The closure of the synagogues as part of the national outbreak response was reportedly a particularly impactful event in this regard. This was explained by the essential position the synagogue holds in the life of Orthodox and Haredi Jewish communities, acting not only as a place for prayer, but also to socialize and stay updated on the latest news in the community and beyond. Yet, the closure of the synagogues and other prayer houses led to a need to organize religious life remotely. This reportedly created specific challenges in adhering to religious rules according to which, in order to have a valid prayer, a quorum of at least ten adult Jewish males (i.e. a *minyan*) must be constituted. Since the *Halacha* requires Jewish men to be able to respond to each other’s prayers in real-life, creative solutions were found to overcome this challenge, as illustrated in the quote below:

*“A lot of [Orthodox] men had moved their prayers from the synagogue to the balconies of their apartments. Meaning hundreds of people were praying from their balconies, and wishing each other courage, to hold on. […] From my bedroom I could hear them on Friday nights, people who cannot see each other, yet they were singing together.”*

[Orthodox member of the Shomre Hadas community]

While several Orthodox respondents mentioned they were struggling with organizing their religious activities during the lockdown, not being able to pray was generally not considered to be in conflict with their Orthodox or Haredi identity in such exceptional times. Different interviewees confirmed that one Jewish principle overrides virtually any other religious rule in times of emergencies: in times of danger everything must be done to preserve life (i.e. *pikuach nefesh*). An Orthodox member of the Machsike Hadas community explained this as follows:

*“The first and most important command in the Torah is ‘take good care of your life’. Even when the synagogues closes, your life comes first. Also other customs and rituals that we are ought to perform, yet haven’t been able to adhere to because of this crisis, that is not with a bad feeling because I was not allowed to do them. The first command is much more important than all others.”*

Although respondents recognized this religious justification for not being able to follow all the religious rules inherent to Orthodox Judaism, some reported that it made them feel uncomfortable or frustrated. These feelings were described as not being able to completely live up to what was expected of them in terms of religious observance. One Haredi member of the Machsike Hadas community phrased this feeling as follows:

*“If you are Jewish, and you observe the rules of Judaism, day in and day out, all 613 of them, then you have the feeling that God is part of your family. If you are not able to do that anymore, you feel like an orphan. It is the feeling as if something existential is missing. You have to be Jewish to understand that frustration.”*

#### Family life and an orthodox Jewish lifestyle

Many respondents from Orthodox and especially Haredi Jewish communities reported experiencing challenges related to family life. As Orthodox Jewish households often tend to be quite large, family composition emerged as a significant theme in different interviews. Usually, respondents explained, there is a sort of ‘natural spread’ of household members over the day, where men go to work or to the synagogue, and children attend school. Yet, during the lockdown period all household members had to stay together inside, putting a strain on many parents, in particular on mothers. Moreover, when going outdoors as a family, some respondents experienced a sense of disapproval from other citizens towards their behavior, mistaking their family activities for illegal social gatherings. One Orthodox participant said:

*“I am a father of thirteen children. We are all locked-up, we cannot even go to the park because my car only has seven seats. Even if we do go out, I see other people looking at us, ‘does he take that many people?’, but they do not understand, they are all part of my family!”*

Many interviewees experienced an impact of the epidemic on the way important Jewish life events and holidays are traditionally celebrated. Usually, there would have been extensive travelling between families living in different parts of the world for occasions such as weddings, funerals, or important Jewish holidays. Especially the celebration of Passover (*Pesach*), one of the major Jewish holidays traditionally celebrated for 8 days in spring time, was particularly impacted by the first lockdown. During this period, many countries had either closed their borders or strongly discouraged international travel. The physical separation of families and community members during traditional and highly symbolic events essentially centered around social gatherings, was described to weigh heavily on community members. This challenged usually applied coping mechanisms during stressful times (e.g. being able to mourn as a group during funerals).

*“Our community [of Haredim] in New York was hit hard…Even I lost my brother-in-law In fact everybody here knows someone who passed away in the epidemic in New York. Yesterday I talked to somebody who lost five people… And he couldn’t go to their funerals. First, because there are too many funerals, and second because we cannot travel. We will remember this crisis for a long time I think…”*

[Member of a Haredi community]

#### Risk perception and stigma

Several respondents felt that the Jewish communities had been negatively portrayed in mainstream and social media, both nationally and internationally. They felt that such media reports often overemphasized the negative aspects of risk and vulnerability of Orthodox Jewish communities to COVID-19, while this was not always in accordance with how they perceived the local reality. Many interviewees did not have the impression that the Antwerp Jewish communities were being disproportionately affected by COVID-19 in terms of cases or fatalities. According to an Orthodox member of the Shomre Hadas community:

*“It is often like that, when a problem emerges in the world, that the Jews are singled out. It was like that with the Plague, and with many others. And like I mentioned, there was an article in the beginning that stated that Orthodox Jews were not adhering to the rules of social distancing and that 500 Jews will die from corona. That can be used as anti-Semitism, those anti-Jewish attitudes: ‘Oh yes, it is because of them that corona is here’.”*

Moreover, a few interviewees reported having experienced negative consequences during the epidemic such as, for instance, ‘over-policing’ of Jewish neighborhoods or they shared stories of overt discrimination and anti-Semitism towards other members of the Orthodox Jewish communities.

*“The communication of the media was a bit against us, like ‘85% of the Jewish community will be infected’. Then other citizens became suspicious, and we have been in a position, in the diamond industry, but also in the streets, that people were avoiding us. And that word, ‘filthy Jew’, appeared again…These are things that have happened, and they shouldn’t be happening.”*

[Traditional member of the Shomre Hadas community]

### Community responses against COVID-19

#### The role of community organizations

Respondents described unequivocally how community organizations had a key role in facilitating the uptake of national outbreak response measures in the Orthodox Jewish communities. One such organization often identified by interviewees was the ‘Crisis Management Team’ (CMT). The CMT was described as a pre-existing volunteer-based organization dedicated to community safety and security, established with the goal to respond rapidly to crisis situations. It involved representatives of the most important community organizations and institutions, including the Rabbis of the two largest Jewish communities (Shomre Hadas and Machsike Hadas). Key informants mentioned how already in the early days of the pandemic, the CMT had made preparations for a local community response through consultation with representatives from official local authorities. They considered reaching out to all members of the community through rapid and community-based communication as the CMT’s most important task during the pandemic. Volunteers therefore operated a telephone ‘hotline’ which community members could call for questions and updated information on COVID-19 and government measures.

*“Together [with volunteers] the first thing we did was opening a call center, in multiple languages. People could hear a daily message from the upper-Rabbi, people could call and speak to live operators and ask questions. Like, ‘what do I have to do with my children?’, and ‘can I go to the grocery store?’. It could be anything, but of course in a language they could understand, English, Yiddish, Hebrew,…”*

[Community leader]

Key informants mentioned the importance of making sure that all communications on outbreak control measures sent out by the CMT were endorsed by the Rabbinate, as it increased the credibility of these messages and therefore also community members’ motivation to adhere to these measures.

*“The upper Rabbi was on board from the very beginning. He understood that something bad was going on and said ‘use me for whatever you need me’. It is not always like that, because the Rabbinate has a clear role, and they know that people will listen to them. So we asked if we [CMT] could communicate in the name of the Rabbi, so that when we said ‘stay at home’, we said it together with the Rabbi.”*

[Community leader]

In addition to the CMT, several respondents identified the *Hatzoloh* as an equally important community-based organization. Key informants explained how *Hatzoloh* constituted a team of certified volunteer paramedics providing first aid upon receiving emergency calls from community members. Their work was described as complementary to official medical emergency services, and participants saw particular benefits of the *Hatzoloh* in mitigating cultural and linguistic barriers to an adequate emergency response.

#### The role of community and religious leaders

Key informants who had a leading role in community organizations reported having strong connections with official authorities from outside the Jewish communities. This contributed, in their opinion, to a rapid communication stream of official outbreak control measures. It also ensured that important cultural factors could be taken into account in the response of local authorities.

*“The police played an important role here, to sensitize people in the very beginning. So not directly fine them, because they [police] understand that the communication does not always happen very fast in our community, because people do not have internet or TV, and they cannot follow the news like everybody else. And also they don’t always speak the language.”*

[Community leader]

All respondents identified the Rabbis as particularly influential figures in Orthodox and Haredi Jewish communities given their position as religious leaders. Rabbis were considered essential and respected authorities to guide community members in matters of *kosher* practices and living according to the *Halacha* (see also Table 1). During this pandemic, they were therefore considered well positioned to overcome feelings of mistrust towards the government, rooted in the historical involvement of local city authorities in the Holocaust.

*“If you want to know about the psychology of why not everybody takes notice of the laws of the federal government, it might be due to the fact that before the war in Europe, the governments were not so friendly towards the Jews, to say the least. And some of the people in town here don’t always trust the federal government and as far as their laws are concerned.”*

[Orthodox member of the Shomre Hadas community]

Rabbis themselves also recognized the influence they exercised on the community, as illustrated by the quote of a Rabbi below:

*“It is not enough that the federal government gives out laws as far as social distancing is concerned because some people might not take notice of it for whatever reason, and therefore it is very important that the Rabbis of the communities show that they back the federal government’s laws and show that we have to take them seriously.”*

Several respondents stressed the impact that video messages sent out by the Rabbinate via social media and online messaging applications had on community members. Although members of Haredi communities mentioned that internet access was often proactively restricted through the instalment of filters, the use of online messaging applications such as Whatsapp in this group has reportedly become more accepted and widely adopted over the past years because of its value in maintaining social connections. Yet, digital literacy was in general considered to be lower in Haredi communities than in other Jewish communities.

*“When people heard that the upper Rabbi himself said ‘this is serious, don’t go to the synagogue. Even I don’t go to the synagogue now’, that had a big impact. And community members instantly knew ‘this has to be serious’. This message has been shared massively. We are a very connected community you know, this news spreads like a running fire.”*

[Orthodox member of the Shomre Hadas community]

Respondents also recognized the leading role Jewish family physicians had during the lockdown period. Many saw preserving life and good health as one of the most important principles of Jewish religion, and Jewish family physicians seemed highly respected in the community for their role in promoting these values. They were mentioned to have actively participated in the spreading of video messages containing health promotion advice.

#### Jewish solidarity and resilience

Respondents described the community responses against COVID-19 as characterized by a strong feeling of internal ‘Jewish’ solidarity which transcended the cultural boundaries between different (Orthodox) communities with differing traditions and degrees of religious observance (as described in Table 1). While this was also reflected in the activities of the previously discussed various community-based organizations, respondents also referred to numerous forms of ‘self-organization’ to stay socially connected during a period of ‘physical distancing’. The use of telephone calls and text messaging to check on family members acquaintances was reportedly widely used. Additionally, participants described several initiatives that were launched via different Whatsapp discussion groups to support the elderly and vulnerable of the Jewish community at large.

*“There was indeed a rapid reorganization with a strong element of solidarity. There were Whatsapp groups, for instance to take care of older community members, to help them with their groceries. I also participated in those.”*

[Orthodox member of the Shomre Hadas community]

Several interviewees situated this solidarity in a historical context by referring to previous crises they had overcome as a people in the past. They reported that this common history had created a particular sense of community resilience, by fostering a feeling of being capable of collectively and successfully mitigating this current crisis as well.

*“We heard stories of our parents about Jewish people when they were stuck behind the Iron Curtain. Stories of our grandparents during World War II, and the Holocaust. Now it was up to us to combat this crisis as well. That feeling was there.”*

[Orthodox member of the Shomre Hadas community]

### Unmet information and prevention needs

#### Information needs

Although many respondents believed that information on COVID-19 reached the majority of Orthodox Jewish community members either through regular information streams (e.g. mainstream media) or through communications delivered by the CMT, many of them also acknowledged potential delays in introducing tailored information messages in mainly Haredi groups. Reasons identified included (1) a need to translate official communications to Yiddish and Hebrew first; (2) the fact that announcements of official communications by the national government often happened on a Friday night, which corresponded to the commencement of Shabbat and hereby complicated the penetration of information into communities strictly adhering to religious rules disallowing the use of electronic devices during Shabbat; and (3) the absence of television, radio, and internet use in many Haredi families.

*“The start of the lockdown was announced on a Friday night. That is a very difficult timing for Jewish religion to still be of any use because that is when Shabbat starts, so there was no time to communicate all those measures to Orthodox Jews and tell them they could not go to the synagogue the day after. It was already too late. If we had known by Thursday evening, or Friday morning, then we might still have been able to do something.”*

[Community leader]

Additionally, several respondents identified a need to provide a clear rationale behind all outbreak control measures communicated by official authorities. Interviewees referred to various Jewish cultures with a common tradition of providing extensive commentaries on written documents, placing interpretation at the core. Several interviewees felt that if community members were not able to develop a clear understanding of why measures were necessary, this would increase barriers towards their uptake. Therefore, translations of official communications to Yiddish and Hebrew needed, in their opinion, to go beyond the mere translation of the measures as such and include their relevant background. A religious framing, appealing to a highly valued Jewish ethos of ‘preserving life at all cost’, was deemed crucial.

#### Attention for religious life

Many Orthodox interviewees perceived a lack of consideration of the needs of religious communities in Belgium during the first lockdown period. While many respondents identified being able to practice their religion as an essential part of the Orthodox Jewish identity, they also reported several barriers to appropriately accommodating religious activities to the context of the lockdown.

*“Because our society has been increasingly secularized over the last years, there is basically no lobby group for religious people. And we are now ‘collateral damage’ because we are still religious, but we are a minority. If there were a larger Catholic group in Belgium, then prayer houses and religious services would have been way higher on the agenda. But it is not a priority, because people are not religious anymore.”*

[Orthodox member of the Shomre Hadas community]

Including representatives of the Orthodox Jewish communities in decision-making spaces of (local) governance structures was identified as a crucial step towards responding to the particular needs of this group.

*“The problem is that they [the government] don’t even know how hard it is for us. […] If they had known, they would have done something. I understand this, because I talked to people of the government. If we had been able to discuss possible solutions with them, I am sure we could have found something.”*

[Orthodox member of the Machsike Hadas community]

Intensifying the collaboration with local city-level authorities could, according to some respondents, lead towards more inclusive policies. Yet, at the same time respondents also identified potential barriers towards effective community engagement at this level due to the heterogeneity of the Orthodox Jewish communities within Antwerp.

*“That has always been a difficult issue. If you have two Jews, there will be three opinions. It will never happen that we, as one community, will be that organized that we can send a representative to a collaboration platform. There are many reasons. Not everybody in the Jewish community wants the same things.”*

[Member of a Haredi community]

## Discussion

This qualitative explorative study sought to contribute to addressing the lack of evidence on experiences of local communities in coping with COVID-19 and related outbreak control measures. We present the case of the Orthodox Jewish communities in Antwerp as it can demonstrate how cultural, religious, and socio-economic factors related to minority populations’ context may impact on the success of local pandemic responses.

We showed that government-issued COVID-19 control measures clearly had an impact on the distinctive communal life of religious minority communities in Belgium. Although it is difficult to isolate religious and cultural factors influencing people’s adherence with official pandemic control measures from broader socio-economic factors, our study reveals specific challenges faced by Orthodox Jewish communities. These challenges were mainly related to organizing religious life in a remote way, and the practice of ‘physical distancing’ in socially strongly connected communities. Our results also provide insights into how these particular communities coped with these challenges by being able to rapidly mobilize community resources which were partially pre-established before the pandemic hit, revealing their resilience. Importantly, the respondents in this qualitative study reflected a diverse sample including different cultural backgrounds and levels of commitment to religious life (from traditionally Jewish up to strictly Orthodox). This offered us an insight into how lived experiences can differ between these sub-groups, yet these cannot be generalized towards the entire Jewish population residing in Antwerp.

The potential vulnerability of Orthodox Jewish communities to COVID-19 has been highlighted in several reports [19, 36, 37]. Especially the strongly connected communities of Haredi Jews were believed to have been disproportionately affected by the pandemic in large urban areas of, for instance, New York and Israel [11, 19]. While we lack official epidemiological data on the impact of COVID-19 on the Antwerp Jewish communities, many respondents in our research did not feel that their communities had been disproportionately affected by the pandemic during the first lockdown period. These perceptions were also in accordance with several media reports stating that, in contrast with previous predictions, the Antwerp Jewish communities seemed to have rather successfully mitigated the potentially devastating impact of a first wave of infections [38]. Despite recent research showing a very high SARS-CoV-2 seroprevalence in an ultra-Orthodox Jewish community in the UK conducted over October–December 2020, the perceived impact of COVID-19 on the physical health of Antwerp Orthodox Jews during the first lockdown period did not emerge as a prominent theme in our data [39]. However, many respondents referred to the indirect effects of the pandemic on the social and religious lives of community members. Since Orthodox Jewish communities can be described in terms of a collective communal, familial, and socially connected culture that values interaction with other community members, public health measures rooted in the concept of ‘physical distancing’ might indeed be experienced as particularly stressful to their members [40].

Interviewees also provided us with some noteworthy elements that might constitute successful community responses against COVID-19. Firstly, respondents appealed to a feeling of internal solidarity which they felt added to the resilience of Jewish communities in coping with the current pandemic. This feeling was expressed in terms of being able to count on a broad social network providing them with material and mental support. While the close social connections within the Orthodox Jewish communities were previously identified as a potential risk factor for acquiring COVID-19, our study seems to suggest a possible protective effect. This confirms the findings from a recent study done among American Orthodox Jews showing that despite a high level of exposure to COVID-19, levels of stress and negative impact of the epidemic were generally low [37]. Besides an association with the positive effects of religious coping, the authors also referred to the possible benefits of a family- and community-centric culture that Orthodox Jews maintain. In addition, a study done among the three major cultural groups in Israel (secular Jews, Arabs, and ultra-Orthodox Jews) found elevated levels of psychological distress among these groups during pandemic times [41]. However, of all the studied groups, the ultra-Orthodox Jews showed the strongest resiliency factors and the lowest levels of psychological distress. A strong sense of coherence was suggested to be the most important explanatory factor for this correlation. The combination of a strong sense of identity, an ethos of mutual help, and the feeling of belonging, have previously been described as buffers in reaching and maintaining good mental health in Orthodox Jewish communities [42]. Similar mechanisms might have been at play in the communities included in our study.

Consistent with previous research, our data show that even in the early phases of responding to a novel infectious disease outbreak, the rapid mobilization of community resources proved to be paramount to facilitate top-down-issued ‘one-size-fits-all’ control measures [43]. The tailoring of such measures to the needs of socio-culturally diverse sub-groups within communities, including addressing language barriers, is a particular expertise that local experts bring to the table. In addition, historically rooted issues of mistrust towards the government were found to have complicated the initial coverage and uptake of control measures in Orthodox Jewish communities. Nevertheless, our research also shows that in the context of an infectious disease outbreak such as COVID-19, this mistrust can be overcome by the involvement of appropriate and trustworthy mediators who bridge the trust and information gap between communities and (local) authorities. We identified community leaders who maintain various connections both within and outside the communities, such as Jewish family physicians and the CMT, as particularly important to act as a bridge between these two worlds. Their engagement contributes to an effective and community-adapted outbreak response by merging scientific knowledge and expertise from outside the community with ‘insider’ knowledge of local sensitivities. Especially the importance of engaging religious leaders in this process has already been clearly established in the literature on previous disease outbreaks [44]. For instance, experiences of the 2013–2016 Ebola outbreak in West Africa highlighted the need for engaging with local and religious leaders and trusted peers to create an environment of trust within the growing climate of suspicion and fear towards governments’ and international NGOs’ motives in the outbreak response [45]. Respondents in our research articulated an equally important role for Rabbis in facilitating the uptake of the government’s COVID-19 control measures. Rabbis did not only mitigate feelings of mistrust towards the government, they also made sure measures were articulated in a culturally appropriate way that had the potential to induce true behavior change. Notably, in some Orthodox Jewish cultures, the adoption of behavior that crosses traditional social or cultural boundaries might not be obtained without the blessing or advice of the Rabbis [15]. This was also reflected in the narratives presented here, stating that all communications sent out by community organizations on COVID-19 should be endorsed by the Rabbinate to increase their uptake.

However, our study also reveals that we still lack understanding of how to reach sustainable involvement of communities and their leaders in decision-making spaces of local governance structures. Such community participation might lead to better ways to capitalize on existing community expertise and resources during health emergencies to foster community-adapted approaches. Future research should therefore continue to focus on documenting best practices of effective community engagement that go beyond passive consultation and actively include community representation in all steps of the outbreak response cycle. A recently conducted evidence synthesis concluded that such knowledge gaps remain especially evident in high-income countries [44]. Yet, the potential benefits of a community-based approach are well-known and reiterated by this current research study. For instance, communicating in ways that are understandable, relatable, and tailored to people’s competencies to interpret them, was pointed out by community members themselves as essential elements of effective risk communication in their community. It is, however, important to note that communities that are not as strongly socially organized and connected as Orthodox Jewish communities, might not be able to come up with innovative systems to streamline such communication efforts themselves. Moreover, heterogeneity within religious groups further complicates effective communication within these communities and risks overlooking the needs of the most vulnerable community members. Describing the highly heterogeneous Jewish communities as a homogeneous ‘risk group’, risks overstating the importance of certain beliefs or practices which may be particular only to a specific group of community members within ‘the Jewish community’. Therefore, there remains a need to build partnerships that engage in a continuous dialogue with different community representatives and integrate a variety of local experiences into a constantly adapted and diversified response. Particular attention in this regard should be paid to engaging with minority populations, as their voices and needs are particularly at risk of otherwise being marginalized [22].

The findings of this study also point towards the risk of stigmatization that comes with the framing of public reporting on specific population groups. The possible negative consequences of such reports on the attitudes towards minority populations might bring to surface persistent and discriminating stereotypes [46]. Monitoring done by the Tel Aviv University showed an increase of 18% of anti-Semitic incidents since the start of the outbreak, and a study done among a non-probability sample of over 2500 adults in the UK showed that almost 20% of participants believed to some degree in conspiracy theories around COVID-19 involving Jews [47–49]. One of the reasons why this stigmatization towards Jewish people in particular is surfacing during this pandemic, might be found in the (re)production of processes of ‘Othering’ [50]. By attributing undesirable or socially discredited behavior to an unknown or – in relation to prejudices and stereotypes – a historically familiar ‘other’, a false sense of assurance or safety might be maintained among the dominant group [51]. Yet during times of social crisis (i.e. in this case a pandemic), these processes can lead to discrimination and blame. To avert this and to mitigate the negative effects of stigmatization, there is a clear need for inclusive communication towards a broader audience.

### Study limitations

There are several limitations to this study. Firstly, the lack of quantitative data stratified by ethnicity limits our understanding of the epidemiological impact of COVID-19 on this population. Secondly, due to the explorative nature of this rapid assessment, we could not explore some complex social and cultural issues more in-depth, such as the narratives of mistrust towards the government. In addition, there are limitations in terms of the sampling. We relied on a mix of purposive and convenience sampling, to learn from the insights of respondents with different profiles and backgrounds, approaching interviewees as experts of their own experiences with the communities. We did not aim to represent and reflect the heterogeneity of Orthodox Jewish communities residing in Antwerp. Despite several efforts, we failed to include Orthodox Jewish women, which has led to a gender bias in our reporting. Reasons for this might be related to cultural sensitivities of having a male researcher, ‘outsider’ to the community, inviting Orthodox Jewish women for a private conversation. Moreover, gender separation is generally more common in Orthodox Jewish communities to be in accordance with the *Halacha*, and clear gender roles might result in less participation of Orthodox Jewish women in community leadership positions [52]. There are also limitations to the digital methods used for online data collection. This excluded the participation of people who do not have a sufficient level of digital literacy, or do not wish to use electronic devices for other reasons. Lastly, the researcher being perceived as an ‘outsider’ to the communities might have yielded sometimes socially desirable or otherwise unreliable answers. Yet, by triangulating the information obtained through different sources, we aimed to overcome some of these challenges.

## Conclusion

Increasing our understanding of locally embedded community responses to COVID-19 can contribute to the development of effective community engagement strategies in the current and in future infectious disease outbreaks. While this study provided us with some very context-specific insights for the Antwerp Orthodox Jewish communities, we identified valuable lessons for developing community-adapted approaches to managing disease outbreaks in different settings.

Firstly, our findings confirm the importance of engaging community and religious leaders in risk communication to facilitate the uptake of government-issued outbreak control measures. This was done through creating culturally appropriate ways of communicating, and involving religious leaders in the dissemination of information to create a feeling of trust. Secondly, community experts can provide important advice on how to build on the social capital of community members in order to rapidly mobilize available resources, appealing to the resilient side of communities. Lastly, the involvement of community representatives in decision-making and governance structures might help to adequately respond to the needs of minority populations, including the mitigation of stigmatization and their potential negative effects on local communities.

The positive effects of developing community-adapted outbreak responses are not limited to the ‘targeted’ communities but may extend to the entire population. Since the COVID-19 pandemic will not be over anywhere until it is over everywhere, effective community engagement during this pandemic will remain a key strategy to benefit public health at large.

## Data Availability

All relevant data supporting our findings are included in this published article. The complete datasets generated and analysed during the current study are not publicly available because they might contain information that could identify other persons, yet additional anonymized data are available from the corresponding author on reasonable request.

## Abbreviations

CAB: Community Advisory Board
CMT: Crisis Management Team
COVID-19: Coronavirus disease 2019
GDPR: General Data Protection Regulation
SARS-CoV-2: Severe Acute Respiratory Syndrome Coronavirus type 2
WHO: World Health Organization
